# Porcine Reproductive and Respiratory Syndrome Virus Reverse Genetics and the Major Applications

**DOI:** 10.3390/v12111245

**Published:** 2020-10-31

**Authors:** Jayeshbhai Chaudhari, Hiep L. X. Vu

**Affiliations:** 1 Nebraska Center for Virology, University of Nebraska-Lincoln, Lincoln, NE 68583, USA; jayeshvet03@gmail.com; 2 School of Veterinary Medicine and Biomedical Sciences, University of Nebraska-Lincoln, Lincoln, NE 68583, USA; 3 Department of Animal Science, University of Nebraska-Lincoln, Lincoln, NE 68583, USA

**Keywords:** PRRSV, reverse genetics, swine viruses

## Abstract

Porcine reproductive and respiratory syndrome virus (PRRSV) is a positive sense, single-stranded RNA virus that is known to infect only pigs. The virus emerged in the late 1980s and became endemic in most swine producing countries, causing substantial economic losses to the swine industry. The first reverse genetics system for PRRSV was reported in 1998. Since then, several infectious cDNA clones for PRRSV have been constructed. The availability of these infectious cDNA clones has facilitated the genetic modifications of the viral genome at precise locations. Common approaches to manipulate the viral genome include site-directed mutagenesis, deletion of viral genes or gene fragments, insertion of foreign genes, and swapping genes between PRRSV strains or between PRRSV and other members of the *Arteriviridae* family. In this review, we describe the approaches to construct an infectious cDNA for PRRSV and the ten major applications of these infectious clones to study virus biology and virus–host interaction, and to design a new generation of vaccines with improved levels of safety and efficacy.

## 1. Introduction

Porcine reproductive and respiratory syndrome virus (PRRSV) is the causative agent of a disease in pigs that was first reported in the United States (U.S.) in 1987 and subsequently in Europe in 1990 [1]. The virus is now circulating in most swine producing countries and is the leading cause of economic losses to the swine industry. PRRSV infects pigs of all ages, but clinical manifestations are more profound in pregnant sows and young pigs. Pregnant sows infected with PRRSV during the last trimester of gestation may result in abortion with stillborn, partially autolyzed, and mummified fetuses while young pigs infected with PRRSV may develop clinical signs including fever, severe dyspnea, anorexia, lethargy, edema of the eyelids, and blue or red discoloration of the ears or hindquarters (reviewed in [2]).

PRRSV is an enveloped RNA virus which belongs to the family *Arteriviridae*, and the order *Nidovirales* [3,4]. Based on their genomic and antigenic diversity, PRRSV isolates are classified into two genotypes: North American (NA) or PRRSV-2 and European (EU) or PRRSV-1, which share only approximately 65% sequence identity [5,6]. The viral genome is a linear, positive sense and single-stranded RNA molecule of approximately 15 kb size which is capped and polyadenylated at its 5′ and 3′ termini, respectively. The PRRSV genome encodes 11 open reading frames (ORFs). ORFs 1a and ORF1b occupy the 5′ proximal 75% of the genome and encode two polyproteins: pp1a and pp1ab. The pp1a and pp1b polyproteins are processed by viral encoded proteases to generate at least 13–16 nonstructural proteins (nsps) responsible for replication and transcription of the viral RNA genomes (Reviewed in [7]). Recently, a new ORF embedded within the nsp2 region (designated ORF trans frame (TF)) was discovered [8]. The ORF2TF is expressed through both −1 and −2 ribosomal frameshifting to produce two additional nsps: nsp2N—a truncated version of nsp2, and nsp2TF—a trans frame fusion protein consisting of the N-terminal two-thirds of nsp2 fused with a 169-aa C-terminal region encoded by the ORF TF [8,9]. The remaining 25% of the 3′ proximal viral genome comprises eight overlapping ORFs: ORFs 2a, 2b, 3, 4, 5, 5a, 6 and 7, that encode eight structural proteins, namely: glycoprotein 2 (GP2), Envelope (E), GP3, GP4, GP5, ORF5a protein, membrane (M) and nucleocapsid (N) protein, respectively. The structural proteins are expressed from a nested set of sub-genomic (sg) mRNAs, each of which carries a common 5′ end leader sequence, referred to as transcription regulatory sequence (TRS), which is identical to the 5′-proximal part of the genome and is co-terminated at its 3′ end. Detailed information on viral replication has been reviewed extensively elsewhere [10]

As with other positive-sense RNA viruses, the PRRSV genome is fully infectious once it is introduced into cells. This provides the basis for the construction of the reverse genetics system for PRRSV. In this review, we describe the approaches to construct a reverse genetics system for PRRSV and its applications to study virus biology, virus–host interaction, and to design a new generation of vaccines with improved levels of safety and efficacy (Table 1).

## 2. Construction of PRRSV Infectious cDNA Clones

There are two strategies to construct a reverse genetics system for PRRSV depending on whether viral RNA or cDNA is used for transfection to rescue the virus. For the RNA-based transfection, the viral cDNA genome is cloned in a plasmid downstream of a bacteriophage RNA polymerase promoter (e.g., T7 or SP6). The cDNA clone serves as the template in an in vitro transcription reaction to produce full-length viral RNA transcripts, which are electroporated or transfected into permissive cells to produce infectious virus. For the DNA-based transfection, the viral cDNA genome is cloned under a eukaryotic polymerase II promoter such as cytomegalovirus (CMV) promoter. In this case, the plasmid containing the viral cDNA genome is transfected directly into the cells without the need of in vitro transcription.

### 2.1. RNA-Based Transfection Approach

**Figure 1:**
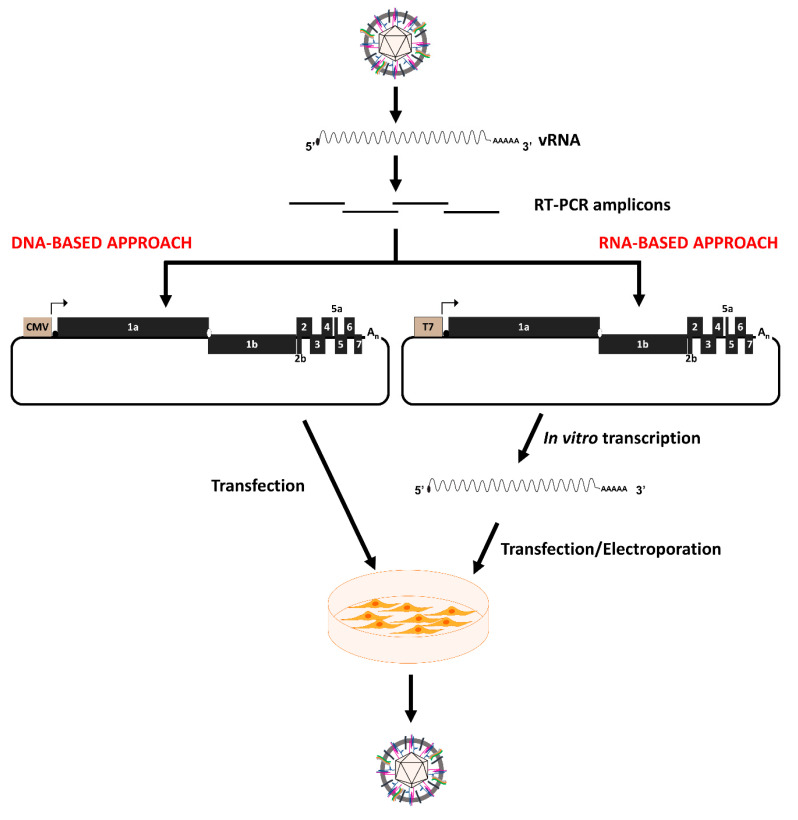
Overview of the approaches to generate an infectious cDNA clone for PRRSV. The PRRSV genomic RNA is isolated and reverse transcribed (RT) to produce complementary DNA (cDNA). Overlapping cDNA amplicons encompassing the full-length viral genome are amplified by reverse transcribed PCR (RT-PCR) and assembled into a bacterial plasmid. For the DNA-based transfection approach, the cDNA genome is cloned downstream of a polymerase II (such as CMV) promoter. The resulting plasmid can be directly transfected into a permissive cell line to rescue the progeny virus. For the RNA-based transfection approach, the cDNA genome is cloned downstream of a bacteriophage (such as T7) promoter. Full-length viral RNA transcript is produced through the use of an in vitro transcription reaction. The viral RNA transcript is transfected or electroporated into a permissive cell line to rescue the infectious virus.

The major steps involved in the construction of an infectious cDNA clone of PRRSV are summarized in Figure 1. Overlapping viral cDNA fragments encompassing the entire viral genome are amplified by reverse-transcription PCR. The fragments are designed in such a way that the overlapping region between two adjacent fragments should contain unique restriction enzyme sites to facilitate cloning. Naturally occurring restriction enzyme sites should be used to minimize the introduction of mutations in the cDNA clone. The cDNA fragments are sequentially assembled to form a full-length viral cDNA genome. Alternatively, overlapping viral genomic cDNA fragments can be assembled without the restriction enzyme digestion by using the Gibson’s DNA assembly approach [83]. Generally, the viral cDNA genome is cloned into a low-copy plasmid because it was initially observed that the viral cDNA genome was not stable when it was cloned into a high-copy number plasmid [84]. However, this may not be a common issue as several PRRSV cDNA genomes have been successfully cloned into high-copy plasmids and those cDNA genomes maintain high-level genetic stability [85,86,87]. A bacteriophage T7 RNA polymerase promoter is inserted immediately upstream of the viral 5′ terminus to facilitate in vitro transcription of the full-length viral RNA genome. A stretch ranging between 21 and 109 adenine (A) residues is incorporated into the 3′ terminus of the cDNA genome to constitute a viral poly-A tail [86,88]. It is not known if the length of the poly-A tail affects the infectivity of the viral RNA genome. Additionally, a unique restriction enzyme site should be incorporated immediately after the poly-A tail to facilitate linearization of the plasmid prior to in vitro transcription to minimize the incorporation of non-viral nucleotides to the viral 3′ end. Finally, silent mutations can be incorporated into the viral cDNA genome to create a new restriction enzyme site that serves as a genetic marker to distinguish the cloned virus from its parental virus [89,90].

To rescue infectious virus from the cDNA clone, the plasmid containing the viral cDNA genome is linearized by digestion with a restriction enzyme incorporated at the 3′ terminus, downstream of the poly-A tail. The linearized plasmid DNA is used as a template for an in vitro transcription reaction to produce 5′-capped full-length viral RNA transcripts, which are then transfected or electroporated into MARC-145 cells to rescue the infectious virus (Figure 1) [38,51,89]. In some instances, the viral RNA transcript is transfected to baby hamster kidney (BHK-21) cells and the supernatant collected from BHK-21 transfected cells containing progeny virus is then used to infect MARC-145 cells to amplify the virus [88,91].

Like many other RNA viruses, PRRSV exists as quasispecies [92]. To ensure that the full-length cDNA clone does not carry any lethal mutations and that the progeny virus derived from the cDNA clone maintains the phenotype of its parental virus, it is important to sequence multiple clones of each amplicon and only clones containing the consensus sequences should be used to assemble the full-length cDNA [89].

In the case of other positive-sense RNA viruses, the addition of non-viral nucleotides to the viral termini might severely affect viral replication [93]. However, the PRRSV viral RNA tolerates the addition of a few non-viral nucleotides at both termini. When the T7 RNA promoter is used for in vitro transcription of the viral RNA, one or two guanosine residues can be introduced between the T7 promoter and the first nucleotide of the viral genome to enhance the in vitro transcription efficiency [89]. This leads to the addition of one or two non-viral nucleotides to the viral 5′ terminus. Similarly, there will be a few non-viral nucleotides added to the poly-A tail of the viral RNA transcripts, depending on which restriction enzyme is incorporated into the 3′ end for linearization. However, the non-viral nucleotides do not seem to severely affect the infectivity of the viral RNA transcripts. Moreover, the non-viral nucleotides are removed from the viral genome of the rescued virus, indicating that the viral RNA polymerase can correct the additional residues at the viral termini during replication [89]. Although the addition of non-viral sequences to the viral genome termini does not affect the recovery of progeny virus, maintaining the authenticity of the viral termini might enhance viral yield [17].

### 2.2. DNA-Based Transfection Approach

The RNA-transfection approach is highly effective, but inconvenient, as it requires an in vitro transcription reaction. To overcome this limitation, Lee et al. introduced the DNA-transfection approach in which the full-length viral cDNA genome is cloned into a bacterial plasmid downstream of a polymerase II promoter [86]. The resulting DNA plasmid is directly transfected into a susceptible cell line where the viral genome is synthesized, and progeny virus is produced. Technically, any polymerase II promoter can be used to drive the synthesis of viral RNA in situ. Thus far, the human cytomegalovirus (CMV) immediate early promoter has been commonly used (Figure 1). It has been suggested that the number of nucleotides between the TATA box and the first nucleotide of the viral genome should be adjusted to 24 residues to ensure that the transcription by RNA polymerase II begins at or very near to the 5′ end of the viral genome [86]. Thus, it is possible to incorporate a T7 RNA promoter between the CMV promoter and the viral genome to create a dual promoter that allows the flexibility of using both DNA and RNA transfection approaches [16]. To ensure the authenticity of the viral termini, hammerhead ribozyme and hepatitis delta virus ribozyme are, respectively, incorporated to the 5′ and 3′ end of the cDNA sequence in the DNA-based infectious clone [67,85]. It has been demonstrated that the DNA-based transfection system produces 10 to 100 fold higher viral titers than the RNA-based transfection system [85,86].

## 3. Application of Reverse Genetics System

### 3.1. Understand the Regulation of Viral RNA Synthesis

Like all RNA viruses, the synthesis of the PRRSV genome and sg RNAs are carried out by virally encoded RNA replicase [10]. Viral nsp1α and nsp1β each contains a papain-like proteinase (PLP) domain, namely PCPα and PCPβ, respectively, that directs the cleavage of nsp1α and nsp1β from the polyprotein (Reviewed in [7]). Inactivation of PCPα activity completely blocks sg mRNA synthesis but does not affect viral genome replication. In contrast, mutations that inactivate PCPβ activity of nsp1β completely block viral genomic and sgRNA synthesis [11]. Similar to nsp1α, nsp12 is not required for viral genomic RNA synthesis but is indispensable for viral sg RNA synthesis [94]. Two Cys residues at position 35 and 79 in nsp12 are cooperatively required for sg mRNA synthesis [12]. Together, these studies clearly demonstrate that genomic and sg RNA synthesis are regulated differently.

It is hypothesized that the PRRSV genome must contain specific sequences or structures known as *cis*-acting replication elements to recruit the viral replicase to the genome [14]. Through the use of a PRRSV-1 infectious clone, a series of deletion mutants at the viral 3′ terminus was constructed [13,14]. It was found that the viral 3′ end contains at least two putative hairpin structures and that base-pairing interaction between these two hairpin structures is critical for viral genomic and sg RNA synthesis [13,14]. Compared to PRRSV-1, the 3′ untranslated region (UTR) of PRRSV-2 contains a stretch of 37 extra nucleotides which can be removed without severely affecting viral replication efficiency [15]. Additionally, the 3′ UTR of PRRSV-2 can be replaced by the counterpart of PRRSV-1, demonstrating the functional compatibility between PRRSV-1 and PRRSV-2 3′ UTRs [15].

Similarly, the 5′ terminus also contains secondary RNA structures that are critical for viral RNA synthesis [16,18]. The 5′ UTR of PRRSV-2 can be replaced by that of PRRSV-1 without affecting viral infectivity but replacement of PRRSV-1 5′ UTR by its counterpart of PRRSV-2 abolishes viral infectivity [18]. The viral 5′ terminus can tolerate up to seven nucleotides’ deletion although removal of these seven nucleotides severely affects virus yield [16,17]. Interestingly, progeny viruses rescued from these mutant viral RNA transcripts quickly regain the deleted sequence, indicating that the 5′ terminus undergoes a strong selection pressure.

A hallmark of the PRRSV genome organization is the overlapping nature of structural genes [10]. Consequently, manipulation of the coding sequence of one gene might also affect the overlapping gene. To overcome this challenge, recombinant cDNA clones were constructed in which overlapping genes were separated and restriction enzyme sites were inserted between the genes [19,20]. The resulting mutant viruses replicate indistinguishably from their parent and these mutant viruses are genetically stable after multiple passages in cell culture, demonstrating that the overlapping nature of the structural genes does not influence viral genomic and sub-genomic RNA synthesis [19,20].

### 3.2. Identification of Essential and Non-Essential Viral Genomic Regions

It appears that all PRRSV proteins are required for the production of fully infectious viral particles. Removal of genes encoding either GP5 or M, the two major structural proteins, from the viral cDNA genome of PRRSV-1 completely abolished viral particle formation [21]. In contrast, removal of the genes encoding the minor glycoproteins GP2, GP3 and GP4 did not affect viral particles formation but the viral particles lacking these minor glycoproteins were noninfectious, presumably due to the defect in viral interaction with the host cell receptor [21]. Similarly, no infectious virus was rescued from cells transfected with a recombinant cDNA clone of PRRSV-2 from which the coding sequence of E protein, GP2, GP3 and GP4 was deleted [22]. Interestingly, infectious virus was rescued when the deletion mutant cDNA clones were transfected to a cell-line stably expressing the glycoproteins [22].

Envelop (E) protein is a small protein encoded by ORF2b that is embedded entirely within ORF2a, encoding GP2 [95]. Accordingly, E protein and GP2 are translated from the same sg mRNA2. The former protein is translated through an alternative translational initiation codon located inside ORF2a [95]. To understand the role of E protein in the viral replication cycle, the translational initiation codon of ORF2b was mutated in a full-length cDNA infectious clone [23]. Viral particles containing genomic RNA were produced from cells transfected with the ORF2b knocked-out cDNA clone. However, the viral particles devoid of E protein were non-infectious, presumably due to a defect in the uncoating and release of the genome into the cell cytoplasm [23]. Mutations of Cys residues or myristylation motif in the E protein do not affect viral infectivity [24,25].

ORF5a protein is a small non-glycosylated structural protein which is expressed from an alternative translation initiation codon of sg mRNA 5, preceding the initiation codon of the major envelope glycoprotein GP5 [96]. PRRSV-2 ORF5a protein contains 2 conserved Cys at position 29 and 30 while PRRSV-1 ORF5a protein contains only one Cys residue at position 30. The protein interacts noncovalently with itself and with the GP4 and E proteins [27]. Both Cys residues in the ORF5a of PRRSV-2 are not essential for virus infectivity [27]. While the biological functions of the ORF5a protein remain largely unknown, this protein was found to be indispensable for viral infectivity [26].

N protein interacts with the viral genome to form the nucleocapsid [97]. Deletion of the gene encoding N protein completely abolishes viral infectivity. However, single-round infectious PRRSV replicon particles can be rescued by using a *trans-*complementation system with a recombinant cell line stably expressing N protein [98]. N protein is a serine phosphoprotein that exists as a homodimer [28]. PRRSV-2 N protein contains three conserved Cys residues at positions 23, 75, and 90, of which Cys_23_ is responsible for protein homodimerization [28,86]. It was reported that substitution of Cys_23_ by Ser completely abolished the infectivity of a PRRSV-2 cDNA clone, presumably due to the disruption of N protein homodimerization [86]. Interestingly, a Cys_90_Ser mutation also abolished infectivity of this PRRSV-2 cDNA clone even though Cys_90_ is not involved in N-N disulfide linkage formation. However, in another study, it was reported that infectious progeny viruses could be rescued from both PRRSV-1 and PRRSV-2 cDNA clones when all Cys residues in the N protein were simultaneously mutated to Ala. The results of this study suggested that noncovalent interactions between N proteins are more important for N protein dimerization during virus particle assembly than cysteine-mediated disulfide linkages [99]. N protein also contains a nuclear localization signal that directs the translocation of N protein to the nucleolus of infected cells [100]. Recombinant cDNA clones devoid of the N protein nuclear localization signal (NLS) are fully infectious but their replication efficiency is severely affected [29,30]. Interestingly, the PRRSV mutants devoid of the NLS are attenuated when inoculated to pigs but they are able to elicit greater titers of neutralizing antibodies than wild-type virus [29,30].

The PRRSV genome encodes for 13–16 non-structural proteins [7]. Nsp2 is the largest non-structural protein and contains multidomain: a small highly variable domain (HV1) followed by a cysteine protease domain (PLP2) in its N-terminal domain, a large highly variable domain in the middle (HV2) and a transmembrane domain (TM) in its carboxyl terminus [32]. The PL2 protease domain possesses both *cis-* and *trans-*cleavage activities, which mediate the self-release of nsp2 from polyprotein pp1a/pp1ab [31]. This process is essential for virus replication as mutations that abolished the PL2 protease activities domain or the cleavage site between nsp2 and nsp3 are detrimental to the virus’s infectivity [31]. A series of mutants containing deletions at different regions of the nsp2 were constructed based on the cDNA clone of the PRRSV-2 strain VR-2332 [32]. This study reveals that the HV1 domain upstream of the PL2 domain (aa 13 to 35) is dispensable for viral replication while the middle of the HV2 domain (aa 324 to 813) tolerates up to 400 aa deletion [32]. Subsequently, multiple PRRSV mutants carrying a deletion in the HV2 region have been successfully generated [58,59,76,101,102,103,104,105]. In some cases, the deletion of a portion of nsp2 enhances viral replication in cell culture [87,103,104]. Besides deletion, nsp2 also tolerates large insertion of foreign genes. This topic will be discussed in more detail below.

Recently, a new ORF, namely ORF TF, was discovered within the coding region of the viral nsp2. This new ORF encodes for a new protein called nsp2TF that is expressed as -2 ribosomal frameshift [8]. The expression of nsp2TF is not required for the virus’s infectivity, however, removal of nsp2-TF expression significantly reduces viral fitness [8].

### 3.3. Study the Significance of N-Linked Glycosylation of Viral Glycoproteins

Glycosylation modification of viral envelope proteins is critical for proper protein folding, viral particle assembly and receptor interaction [106]. PRRSV particles contain four glycoproteins: GP2, GP3, GP4 and GP5. Based on their relative abundance on viral virions, GP2, GP3 and GP4 are considered minor glycoproteins while GP5 is considered the major envelope glycoprotein [107,108]. The number of potential N-linked glycosylation sites in GP2, GP3 and GP4 of both PRRSV-1 and PRRSV-2 are two, seven, and four, respectively [33,109,110]. PRRSV-1 GP5 contains 2 N-linked glycosylation sites [110] while PRRSV-2 GP5 contains three or four sites [37,38]. PRRSV isolates that naturally lack N-glycosylation sites have been reported [38,111].

The significance of N-glycosylation of GP2 and GP5 has been studied in both PRRSV-1 and PRRSV-2, while the influence of N-glycosylation of GP3 and GP4 has only been reported for PRRSV-2 [33,34,35,36,37,38]. None of the individual N-glycosylation sites in GP2, GP4 and GP5 are essential for the formation of infectious viral particles of both PRRSV-1 and PRRSV-2 [33,34,35,36,37], except the N-linked glycosylation site Asn_44_ of GP5 whose disruption severely affects viral infectivity [36,37]. The influence of N-glycosylation of GP3 is controversial. One study reported that glycosylation at position Asn_42_, Asn_50_ and Asn_131_ of GP3 is necessary for infectious virus production [33], while the other study reported that none of the individual glycosylation sites in GP3 have a vital effect on the production of infectious virus [35]. In the former study, Asn residues were substituted to Ala, while in the latter study, the Asn residues were substituted to Ser. The nature of the amino acid substitutions used to disrupt the N-glycosylation sites in PRRSV glycoproteins seems to be critical for the recovery of infectious virus [35]. It is possible that the discrepancy might be due to the strain effects as these two studies were conducted using two different PRRSV strains.

Glycosylation of the viral envelope proteins is one of the mechanisms for viruses to escape neutralizing antibody, a phenomenon known as glycan shielding (reviewed in [112]). In the case of PRRSV, the removal or insertion of N-linked glycosylation sites in GP5, respectively, enhances or decreases the viral susceptibility to antibody neutralization [34,37,38]. Therefore, PRRSV mutants lacking glycosylation sites in GP5 have been proposed to be used as a vaccine immunogen to immunize pigs to enhance the stimulation of neutralizing antibodies [113]. On the other hand, glycosylation sites in minor glycoproteins might not be significant for viral immune evasion. Removal of N-glycosylation sites in GP2 and GP4 of PRRSV does not alter the virus’s susceptibility to antibody neutralization [33,35]. In one study, it was reported that a PRRSV strain naturally lacking a glycosylation site at position N131 in GP3 was highly susceptible to antibody neutralization [38]. Reintroduction of the N-glycosylation site to this position significantly reduced the virus’s susceptibility to antibody neutralization, demonstrating the significance of the N-glycosylation at this site in neutralizing antibody recognition [38]. However, it was reported in another study that the removal of each individual N-glycosylation site in GP3 of a different PRRSV strain did not significantly alter the virus’s susceptibility to antibody neutralization [35]. Again, we believe that the different PRRSV strains used in those studies might account for the inconsistent findings.

### 3.4. Identify Viral Determinants of Cell Tropism

PRRSV mainly infects cells of the monocyte/macrophage lineage (reviewed in [114]). To identify viral proteins that are responsible for the viral cell tropism, a series of the chimeric viruses has been constructed by swapping viral proteins between PRRSV and equine arteritis virus (EAV), a prototype virus of the family *Arteriviridae*. Using EAV’s infectious cDNA clone as the backbone, chimeric viruses were generated in which the ectodomains of the two major envelope proteins, GP5 and M, were replaced by the corresponding sequences of PRRSV [115,116]. The resulting EAV/PRRSV chimeric viruses failed to infect porcine alveolar macrophages (PAMs), the susceptible cells for PRRSV [19]. In a reciprocal study, the ectodomain of PRRSV M protein was replaced by the corresponding sequence of two related viruses: EAV and lactate dehydrogenase virus (LDV). The PRRSV chimera carrying M ectodomain of EAV or LDV maintained the infectivity in PAM [19]. These two studies demonstrated that GP5 and M ectodomain of PRRSV are not the viral determinants of cell tropism. Using PRRSV infectious clone as the backbone, a chimeric virus was generated by swapping PRRSV minor envelope proteins (E, GP2, GP3 and GP4) with the corresponding proteins of EAV. The resulting PRRSV/EAV chimeric virus lost its ability to infect PAMs but gained the ability to infect cell lines normally susceptible to EAV [39]. Thus, the minor envelope proteins are the main determinants of the virus cell tropism.

Recently, a series of PRRSV mutants bearing different deletions in the nsp2 region were generated to study the role of this protein in viral transcription and replication [43]. It was reported that deletion at the hypervariable region (amino acid 323 to 521) significantly impaired the virus infectivity in PAMs although this mutant virus still replicated efficiently in MARC-145 cells [43], a monkey kidney cell line widely used to propagate the virus in vitro [117]. Thus, nsp2 is one of the viral determinants of PRRSV tropism.

Primary PRRSV field isolates often need to be adapted for several passages before they can replicate efficiently in MARC-145 cells. Several PRRS modified live virus (MLV) vaccines have been made by consecutively passaging the virus in MACR-145 cells (reviewed in [118]). Through swapping genes between MARC-145 adapted and non-adapted PRRSV isolates, it was found that genetic variations in GP2 and GP3 seem to be accountable for the virus’s adaptation to this cell line [40]. Additionally, site-directed mutagenesis has been used to modify specific amino acid residues in E, GP2 and GP3 to demonstrate their significance in enhancing virus replication in MARC-145 [41,42,44].

### 3.5. Characterize Viral Targets for Antibody Recognition and Neutralization

The early identification of one neutralizing epitope in the GP5 ectodomain has led to the assumption that GP5 is the main target of virus-neutralizing antibody [119]. A comparative analysis of a panel of 69 GP5 sequences from the PRRSV field isolates in conjunction with their susceptibility to antibody neutralization revealed five putative variable sites in GP5 that differentiate susceptible and resistant PRRSV isolates [45]. Site-directed mutagenesis was employed to substitute amino acid residues within these five sites in the VR-2332 strain. The results showed that three putative sites (aa 32–34, 38–39, and 57–59) located in the GP5 ectodomain significantly influenced the susceptibility of the mutant viruses to neutralizing antibody [45]. These antigenic sites might serve as genetic markers to predict the degree of susceptibility to antibody neutralization.

Besides GP5, other envelope proteins, especially the minor glycoproteins, might also be important for antibody neutralization since they interact with CD163 [109], the key receptor of PRRSV infection [120]. To assess the relative contribution of each envelope protein to the virus’s susceptibility to antibody neutralization, a set of chimeric viruses were generated by replacing envelope proteins of VR-2332, the prototype strain of PRRSV-2, with the corresponding proteins of antigenically distinct PRRSV strains [46]. The results showed that individual replacement of GP3 or GP5 partially reduced the virus’s susceptibility to antibody neutralization by VR-2332 antisera (e.g., homologous) but increased the virus’s sensitivity to neutralization by antisera raised against the donor strains from which the GP5 was used to introduce the virus into the VR-2332 backbone. In contrast, simultaneously replacing ORF3-6 of VR-2332 with the corresponding ORFs of JA142 completely reduced the susceptibility of the VR-2332 antiserum while increasing its susceptibility to the JA142 antiserum [46]. In a subsequent study, an inter-species PRRSV chimeric strain was generated by simultaneously replacing ORFs 2–4 of the PRRSV-1 strain SD01–08 with the corresponding gene of the PRRSV-2 strain FL12. While the PRRSV strain SD01-08 was completely resistant to neutralization by the FL12 antisera, the chimeric virus bearing ORFs 2–4 of FL12 was partially susceptible to neutralization by FL12 antisera [47]. Collectively, these studies demonstrate that all envelope proteins are involved in virus neutralization.

Several PRRSV antibody-escaped mutants were generated by propagating the PRRSV strains in the presence of broadly neutralizing antisera [48,49]. Sequence analysis revealed multiple mutations in the structural genes of the antibody-escaped mutants. Through the use of site-directed mutagenesis, it was demonstrated that amino acid substitutions at positions 102 and 104 in GP5 or deletion of Tyr_10_ in the M protein are associated with antibody resistance [48,49].

Although PRRSV-1 and PRRSV-2 share only approximately 65% sequence identity [5], there are a few monoclonal antibodies that recognize both PRRSV species [6]. Of these broadly reactive monoclonal antibodies, SDOW17, recognizing a conformational epitope in N protein, is widely used to detect PRRSV-infected cells. However, there is a small percentage of PRRSV isolates that are not recognized by SDOW17 MAb. Sequence analysis of one of these PRRSV strains (designated IVI-1173) revealed a substitution from Thr to Ala at position 90 of the N protein. Converting Ala_90_ to Thr in the N protein of PRRSV isolate IVI-1173 restored the reactivity of this PRRSV isolate to SDOW17. Conversely, a Thr_90_ to Ala mutation of a PRRSV isolate RVB-581 abolished the reactivity of this PRRSV isolate to mAb SDOW17 [50]. Thus, Thr_90_ of N protein is a key residue of the conformational epitope recognized by mAb SDOW17.

### 3.6. Identify Viral Determinants of Virulence

A reverse genetics system has been employed to identify the molecular basis of PRRSV virulence, which is important for the rational design of live-attenuated vaccines. The main approach is to exchange gene fragments between wild-type virulent PRRSV strains and MLV vaccine strains. In one study, a series of the chimeric virus was constructed by exchanging gene fragments between an MLV vaccine strain and a virulent PRRSV strain. This study led to the identification of genes encoding nsp 3–8 and GP5 as the main determinants of PRRSV virulence [51]. In another study, two chimeric PRRSV mutants were constructed by separately exchanging the gene fragments containing nonstructural genes (5′UTR and ORFs 1a and 1b) or structural genes (ORFs 2–7 and 3′ UTR) of an MLV vaccine strain with the corresponding gene fragments of a virulent strain. Both chimeric PRRSV mutants were attenuated when they were inoculated to pigs, suggesting that virulence factors reside in both nonstructural and structural genes [52]. This finding led to a notion that new MLV PRRSV vaccines could be molecularly generated by swapping the structural genes of an emerging virulent PRRSV strain into the backbone containing non-structural genes of a currently available MLV vaccine [52]. The rationale is that the nonstructural genes of an MLV vaccine strain will confer attenuation while the structural proteins of an emerging strain will provide heterologous protection since they match the genetics of contemporary PRRSV strains.

In 2006, an atypically high virulent PRRSV strain (designated HP-PRRSV) emerged and become pandemic in China [121]. The HP-PRRSV strain carries a unique 30-amino-acid deletion in its nsp2-coding region as compared to classical PRRSV strains, suggesting that the 30-amino-acid deletion in nsp2 of HP-PRRSV might be responsible for its unprecedented levels of virulence [121]. However, replacing the nsp2 region containing the 30-amino-acid deletion with the corresponding sequence of a low virulent strain did not significantly alter the virulence of the HP-PRRSV strain, indicating that the 30-amino-acid deletion in nsp2 is not associated with the enhanced virulence of the HP-PRRSV [122]. Subsequently, a series of chimeric PRRSV mutants were generated by reciprocally exchanging gene fragments between the HP-PRRSV strain and a low virulent strain. This study led to the finding that nsp9 and nsp10 together are responsible for the fatal virulence of HP-PRRSV [53]. Recently, it was reported that two amino acids at positions 519 and 544 in nsp9 of the HP-PRRSV might be involved in its enhanced pathogenicity [54].

### 3.7. Eliminate the Viral Immunosuppression

PRRSV is well characterized for its ability to suppress the host innate immune responses, especially the type-I interferons (IFN). Viral proteins involved in innate immune suppression and the signaling pathways have been discussed in several reviews [7,123,124]. In this section, we will focus on research that employed reverse genetics to modify the viral genome to subvert the viral suppression of innate immunity. The general approach is to employ site-directed mutagenesis to replace or remove critical amino acid residues residing within viral proteins that are involved in the innate immune suppression. Manipulation of viral protein domains involved in innate immune suppression is often lethal to virus replication. However, there are a few mutants with a relief of innate immune suppression that have been successfully generated.

Site-directed mutagenesis was used to introduce mutations into different regions of nsp1β including the stretch of five residues (16–20), the SAP domain and the conserved motif GKYLQRRLQ [55,56,57]. The resulting mutants induced significantly higher levels of type I IFN while replicating less efficiently in vitro and in vivo as compared to corresponding wild-type PRRSV strains. The mutations were unstable when they were inoculated to pigs and the revertant virus quickly regained the ability to suppress IFN production, clearly indicating that the virus is under a strong selection pressure to maintain its ability to suppress the type-I IFN [55,125].

Nsp2 contains a cysteine protease (PLP2) domain that belongs to the ovarian tumor (OTU) protease family of deubiquitinating (DUB) enzymes (reviewed in [7]). The nsp2 OTU domain interferes with the NF-kB signaling pathway to suppress the induction of type-I IFN [126]. Besides, this protein also inhibits interferon-stimulated gene (ISG) 15 production and ISG15 conjugation to cellular proteins [127]. A panel of PRRSV mutants were generated either by substitution of amino acid residues within the OTU domain or deletions and substitutions at the N-terminal border of the PLP2-DUB domain. Mutations that significantly impaired the ability of nsp2 to inhibit NF-kB activation or ISG15 expression were lethal to virus replication while mutations with a slight reduction in NF-kB inhibition or ISG15 expression resulted in viable recombinant virus [126,127].

Successive passaging of a HP-PRRSV strain in MARC-145 cells resulted in an attenuated PRRSV strain that contains a spontaneous 88-amino-acid deletion in the nsp2 HV2 region [128]. Subsequently, reverse genetics was used to introduce an 88-aa deletion into the nsp2 of a HP-PRRSV strain. The resulting PRRSV mutant carrying this 88-aa deletion showed the enhanced ability to induce type I interferon (IFN-α and IFN-β), and other chemokines [58]. Besides its role in IFN suppression, nsp2 seems to be able to induce IL-1β, IL-6, and TNF-α [59]. Deletions of aa residues 323–433 and 628–747 in the nsp2 of a HP-PRRSV strain yielded fully infectious PRRSV mutants with reduced ability to induce inflammatory cytokines in infected cells [59].

Nsp11 is a strong IFN antagonist [129]. The protein contains a conserved endoribonuclease (EndoU) domain in its carboxyl terminus that is important for the protein suppression of IFN [130]. Using a PRRSV-2 infectious clone, seven single amino acid substitutions were introduced into the EndoU domain to eliminate its suppression of IFN. However, none of the EndoU knock-out mutants yielded infectious progeny virus, indicating that the EndoU of activity is critical for viral replication [130].

While IFN suppression is a common phenotype of PRRSV, there are a few PRRSV strains that were found to be able to induce IFNs [61,131]. The IFN-inducing PRRSV strains provide a unique opportunity to identify viral genetic elements associated with the IFN induction. Recently, a fully synthetic PRRSV strain (designated PRRSV-CON) was generated (discussed in detail in Section 3.8.2) that induces IFN both in cell culture and in infected pigs [61,70,132]. By reciprocally swapping gene fragments between PRRSV-CON and FL12—a naturally occurring strain suppressing IFN [129]—it was discovered that the IFN-inducing phenotype of PRRSV-CON mapped to the 3.3 kb genomic fragment encoding three viral nonstructural proteins: nsp1α, nsp1β and the N-terminal part of nsp2 [61]. A2MC2 is a naturally occurring PRRSV-2 strain capable of inducing IFNs in cell culture [131]. Swapping gene fragments between A2MC2 and VR-2385—an IFN-suppressing strain—revealed that the middle two fragments ranging from nucleotide 4545 to 12,709 of the A2MC2 genome were associated with the IFN inducing phenotype [60].

Some PRRSV strains were reported to induce IL-10 that contribute to the virus’s ability to suppress the host immune system (reviewed in [133]). Transfection of porcine macrophage with viral N protein of a PRRSV-2 strain significantly upregulated IL-10 mRNA expression [62]. Manipulation of residues 33–37 in the N protein resulted in recombinant PRRSV mutants that induce significantly lower levels of IL-10 production in infected monocyte-derived dendritic cells than the corresponding wild-type PRRSV strain [62].

PRRSV nsp1α and nsp1β were found to suppress tumor necrosis TNF-α production at both transcriptional and post-transcriptional levels [63]. Two PRRSV mutants with mutations at nsp1α Gly_90_ or nsp1β residues 70–74 were generated. These mutant viruses replicated less efficiently but induced significantly higher levels of TNF-α than the corresponding wild-type PRRSV strain [63].

### 3.8. Improve Vaccine Safety and Efficacy

Vaccines against PRRSV have been commercially available since 1994. MLV vaccines are preferred over inactivated vaccines as the former are proven to be more effective (reviewed in [134]). PRRSV strains are attenuated by continuously passaging them in non-host cell lines such as MARC-145, a monkey kidney cell line, or BHK-21 stably expressing CD163, a receptor for PRRSV infection [118]. Major limitations of the currently available MLV vaccines include the potential risk of reversion to virulence, inadequate levels of heterologous protection, and the lack of DIVA marker. Reverse genetics has been used to improve the safety and efficacy of PRRSV vaccines.

#### 3.8.1. Molecular Attenuation

Two different approaches have been used to molecularly attenuate PRRSV strains. In the first approach, the GP5 coding sequences of genetically divergent PRRSV strains were molecularly bred by DNA shuffling, and the shuffled genes were cloned into the backbone of a PRRSV infectious clone [64]. In the second approach, computational programs were employed to replace adjacent pairs of original codons of the PRRSV genes with the pairs of codons that are least frequently used; the process is known as codon-pair deoptimization [135]. Several experimental MLV vaccines have been generated through deoptimization of codon-pairs in nsp1, nsp9 and GP5 of different PRRSV strains [65,66,67]. The reported results are promising as the resulting PRRSV mutants were attenuated while still maintaining the ability to stimulate protective immunity when inoculated into pigs.

#### 3.8.2. Improve Heterologous Protection

The profound genetic diversity among PRRSV isolates poses a significant challenge to the development of broadly protective vaccines [134]. Thus, the major goal of PRRSV vaccine design is to reduce the genetic dissimilarity between the vaccine immunogen and contemporary PRRSV isolate circulating in the field to enhance the antigenic match. Multiple chimeric MLV vaccine constructs have been generated by replacing the structural genes of a well-characterized PRRSV strain with the corresponding sequences of contemporary isolates circulating in the swine herds, with the expectation that the genetic match in the structural genes between the chimeric vaccine constructs and the field PRRSV strains will enhance protection [52,136,137]. Alternatively, DNA shuffling technology has been employed to generate mosaic gene fragments containing the genetic sequence of multiple genetically diverse PRRSV strains. The mosaic gene fragments were then cloned into the backbone of an MLV vaccine strain. The resulting recombinant PRRSV strains carrying the mosaic structural genes induced broader levels of neutralizing antibody and conferred partial cross-protection when they were used to immunize pigs [64,68,69]. Recently, a synthetic PRRSV strain was generated based on a chemically synthesized cDNA genome [70]. Specifically, a large set of full genome sequences of PRRSV was used to generate a consensus cDNA genome sequence which was chemically synthesized and cloned into a bacterial vector. Subsequently, reverse genetics was used to rescue fully infectious virus from the synthetic cDNA clone. The resulting synthetic PRRSV strain possesses the same characteristics as naturally occurring PRRSV strains in regard to cell tropism and pathogenicity. Unlike naturally occurring strains, the synthetic PRRSV strain induces type-I interferon instead of suppressing these cytokines [61]. Importantly, it was demonstrated that the synthetic strain conferred exceptional levels of cross-protection against heterologous PRRSV strains [70].

#### 3.8.3. Improve Immune Response

Several laboratories have been exploring the possibility of using immunomodulatory molecules as a biological adjuvant to boost the host immune responses to an MLV PRRS vaccine. The immunomodulatory molecules were inserted into the PRRSV genome in a form of an extra transcriptional unit (discussed in detail in Section 3.9). When the recombinant PRRSV mutants infect the target cells, the immunomodulatory molecules will be expressed which will exert their biological effects on the host immune system. Thus far, PRRSV mutants carrying genes encoding different isotype of IFN, granulocyte macrophage colony-stimulating factor (GM-CSF) and interleukins have been constructed [71,72,73,74]. While the recombinant viruses carrying the immunomodulatory molecules elicit enhanced levels of T-cell responses, they do not confer significantly better protection against challenge infection than the parental strains [71,72,73].

#### 3.8.4. Develop DIVA Marker Vaccines

DIVA marker vaccines are defined as vaccines that lack at least one antigenic component, the so-called serologic marker antigen, when compared to the corresponding wild-type viruses [138]. Therefore, animals naturally infected with wild type viruses, but not those infected with DIVA vaccines, should develop antibodies specific to the marker antigen. Consequently, a differential diagnostic assay that is developed based on the marker antigen can be used to detect naturally infected animals within the vaccinated population [138,139]. DIVA vaccines have been proven to be an important tool for the control and eradication of endemic diseases [139,140]. The PRRSV genome is compact, and all its genes are essential for the viral life cycle. Thus, the main approach to develop DIVA vaccines against PRRSV is to remove a small portion of the viral gene or an immunodominant epitope which, in turn, is used as the diagnostic antigen for generation of the differential test.

Several DIVA vaccine candidates have been generated for both PRRSV-1 and PRRSV-2 through deletion of a coding sequence of immunodominant epitopes within the nsp 2 region [75,76,101]. Pigs vaccinated with the resulting DIVA vaccine candidates do not develop antibodies directed against the deleted nsp2 epitopes while they still mount an immune response against other viral proteins [75,76]. One major drawback of the nsp2-epitope deleted mutants, in the context of a DIVA vaccine, is that the differential peptide-based ELISA used in conjunction with the DIVA vaccines is not very sensitive, mainly due to the substantial genetic variation of this gene [141]. Subsequently, another DIVA vaccine candidate was generated through elimination of a conserved B cell epitope (so-called epitope M-201) located within the carboxyl-terminal of the M protein [77]. Site-directed mutagenesis was used to eliminate the immunogenicity of this epitope. Accordingly, an inhibition ELISA was used to serologically differentiate pigs vaccinated with this M-201 epitope mutant from those that were experimentally infected with wild-type PRRSV strains [77].

### 3.9. Viral Vector

**Figure 2:**
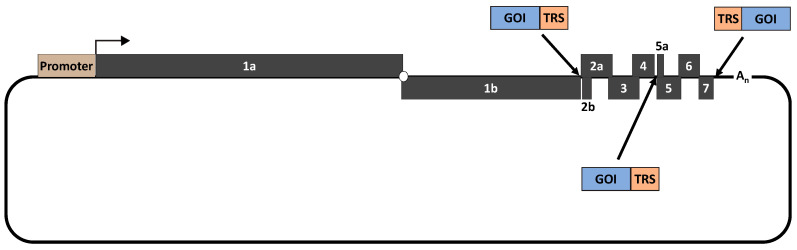
Schematic representation of the potential locations where a gene of interest (GOI) can be inserted into the PRRSV genome. When a GOI is inserted between ORF1b and ORF2a or between ORF4 and ORF5a, its expression will be driven by a transcription regulatory sequence (TRS) located in ORF1b and ORF4, respectively. Consequently, an additional TRS needs be incorporated to the GOI 3′ terminus to drive the expression of ORF2a and ORF5. When the GOI is inserted between ORF7 and the 3′UTR, the TRS needs to be incorporated upstream of the GOI to drive its expression.

PRRSV can be used as a viral vector to deliver a foreign gene of interest (GOI). There are three potential locations where a GOI can be conveniently inserted into the PRRSV genome: between ORF1b and ORF2a, between ORF4 and ORF5a and between ORF7 and 3′UTR (Figure 2) [78,79,80,142,143,144]. Thus far, the intergenic junction between ORF1b and ORF2a is the most commonly used location to insert a GOI. The Capsid protein of porcine circovirus type 2 (PCV2) was inserted to the intergenic junction between ORF1b and ORF2a [78,79]. Pigs vaccinated with a recombinant PRRSV strain containing the PCV2 Cap gene elicited antibodies against both PRRSV and PCV2 Cap protein [79]. In another study, a live attenuated PRRSV strain was used as a vector to deliver two different vaccine antigens: swine influenza virus hemagglutinin (HA) gene and PCV2 cap [80]. In this study, pigs vaccinated with the resulting recombinant PRRSV mutant were modestly protected against challenge infection with either SIV or PCV2.

The genetic instability of the GOI represents a major challenge for using PRRSV as a live viral vector. It was reported in one study that the PCV2 cap gene was deleted from the PRRSV genome at passage 1 after the virus was rescued from the infectious cDNA clone [78]. However, the genetic instability of the GOI seems to depend on the nature of the GOI. When the GFP gene was inserted into a PRRSV genome in the same fashion as the PCV2 cap gene, it was found that the GFP gene was genetically stable after the recombinant virus was passaged in MARC-145 cells for 37 passages [79].

### 3.10. Insert Marker Proteins to Track Viral Protein Translocation and Viral Infection

Recombinant PRRSV mutants expressing epitope tags or reporter genes have been generated by a fusion of epitope tags or reporter genes in-frame into the nsp2, as this viral protein can tolerate large deletion or insertion [32,76,90,145]. Besides, small epitope tags can be fused in-frame into the N protein [146]. The generation of recombinant PRRSV strains expressing reporter genes has provided a powerful tool to study viral protein localization and trafficking, and detection of viral infection [74,81,82]. Using PRRSV mutants carrying a myc-tagged nsp2, it was demonstrated that nsp2, previously known as non-structural viral protein, is indeed incorporated into viral particles [81]. In another study, a recombinant PRRSV mutant containing eGFP-tagged nsp2 was used to infect permissive cells, and a live-imaging system was used to track the intracellular movement of GFP-nsp2 protein [82]. This study revealed that PRRSV utilizes the host cell cytoskeletal machinery inside nanotubes for efficient cell-to-cell spread and, therefore, escapes the effects of virus-neutralizing antibodies.

As discussed above, the genetic instability of the foreign genes inserted into the PRRSV genome has been commonly observed. PRRSV mutant viruses carrying the eGFP gene inserted in the nsp2 region quickly lost a portion of the GFP gene after 3–7 passages in cell culture [32,76,90,145]. The locations where the GFP gene is fused into nsp2 seem to affect the stability of the gene [145]. Furthermore, the removal of a portion of the nsp2 coding sequence before insertion of the eGFP gene might enhance the stability of this gene [76,145]. Even though the reporter gene is unstable, the mutant carrying a reporter gene is still useful for the short term study of intracellular trafficking of the viral protein [82].

## 4. Conclusions

A large number of infectious cDNA clones of PRRSV has been generated. The availability of reverse genetics systems has made it possible to genetically modify the viral genome at precise locations. Common approaches to manipulate the viral genome include site-directed mutagenesis, deletion of viral genes or gene fragments, insertion of foreign genes, and swapping genes between PRRSV strains or between PRRSV and other members of the *Arteriviridae* family. Reverse genetics has been employed to elucidate the biological functions of viral proteins, identify viral determinants of virulence and cell tropisms, unravel the viral mechanisms to evade the host immune system and assist in the rational design of a new generation of PRRSV vaccines with improved levels of safety and efficacy. Potentially, PRRSV can be used as a viral vector to deliver foreign genes of interest, especially genes of swine pathogens. This may lead to the development of multivalent vaccines that can be used to control PRRSV and other swine pathogens.

## Figures and Tables

**Table 1:** Ten important applications of PRRSV reverse genetics.

Significance/Important Findings/Discovery/Research Outcome	References
1. Understand the regulation of viral RNA synthesis	
Differential regulation of viral genomic and sg mRNA synthesis by nsp1α, nsp1β and nsp12.	[11,12]
5′ and 3′ UTR contains secondary RNA structures critical for viral genomic and sg RNA synthesis.	[13,14,15,16,17]
5′ and 3′UTR of PRRSV-1 and PRRSV-2 are functionally compatible.	[15,18]
Overlapping coding sequences in the viral structural region can be separated.	[19,20]
2. Identification of essential and non-essential viral genomic regions	
GP5 and M proteins are indispensable for virion assembly.	[21]
Minor GPs are dispensable for viral particle formation but crucial for infectivity.	[21,22]
E protein possesses ion-channel activities required for viral uncoating.	[23]
Cys residues and myristylation motif in the E protein are not essential for viral infectivity.	[24,25]
ORF5a is indispensable for virus viability and Cys residues in ORF5a are not essential for viral infectivity.	[26,27]
N protein disulfide linkage and NLS are essential for viral replication fitness.	[24,28,29,30]
Essential and non-essential region of nsp2 replicase protein.	[31,32]
Discovery of novel nsp2 TF and its role in virus infectivity.	[8]
3. Understand the significance of N-linked glycosylation of viral glycoproteins	
Individual N-linked glycosylation sites in GP2, GP3 and GP4 are not essential for the formation of infectious viral particles.	[33,34,35,36]
N-linked glycosylation site in GP5 at location 44 is critical for viral infectivity.	[36,37]
N-linked glycosylation in GP3 and GP5 affects viral susceptibility to antibody neutralization.	[35,37,38]
4. Discover viral determinants of cell tropism	
The minor envelope proteins, but not GP5 and M ectodomain, are the major determinants for PRRSV infectivity in PAMs.	[39,40,41,42]
Nsp2 might contribute to viral infectivity in PAMs.	[43]
Mutations in GP2 and GP3 are associated with the enhanced viral replication in MARC-145 cells.	[40,41,42,44]
5. Characterize viral targets for antibody recognition and neutralization	
These antigenic sites in GP5 might serve as genetic markers to predict the degree of susceptibility to antibody neutralization.	[45]
Minor GPs are targets of neutralizing antibodies.	[46,47]
Tyr_10_ in M is associated with antibody neutralization escape.	[48,49]
Thr90 in N protein is aa of the epitope recognized by mAb SDOW17.	[50]
6. Identify viral determinants of virulence	
PRRSV virulence is multigenic.	[51,52]
Nsp9 and 10 are associated with the highly virulent nature of HP-PRRSV.	[53,54]
7. Eliminate viral immunosuppression	
Generation of nsp1β mutants that induce significantly higher levels of type I IFNs.	[55,56,57]
Deletions of different regions of nsp2 of HP-PRRSV strain enhance the virus’s ability to induce type I IFNs while reducing its induction of inflammatory cytokines.	[58,59]
Identification of viral genomic regions associated with a few PRRSV strains that naturally induce IFNs.	[60,61]
aa residues 33–37 of the N protein associated with the viral induction of IL-10.	[62]
Generation of PRRSV mutants that induce high levels of TNF-α.	[63]
8. Improve vaccine safety and efficacy	
Molecular attenuation of PRRSV strain by DNA shuffling and codon pair-deoptimization.	[64,65,66,67]
Improve heterologous protection by DNA shuffling and by de novo synthesis of a new PRRSV strain carrying a consensus genome.	[64,68,69,70]
Improve immune response by insertion of immunomodulators.	[71,72,73,74]
Development of DIVA vaccines by removing dominant B cell epitopes located in the nsp2 and M proteins.	[75,76,77]
9. Viral vector	
Use PRRSV as a viral vector to express PCV2 capsid or dually express PCV2 cap and swine influenza HA protein.	[78,79,80]
10. Insert marker proteins to track viral protein translocation and viral infection	
Insertion of a myc-tag sequence to nsp2 to demonstrate this nsp is incorporated into the viral particle.	[81]
Insertion of GFP to nsp2 to track the intracellular movement of this protein.	[82]
